# Anthracene biodegradation capacity of newly isolated rhizospheric bacteria *Bacillus cereus* S_13_

**DOI:** 10.1371/journal.pone.0201620

**Published:** 2018-08-02

**Authors:** Nadia Bibi, Muhammad Hamayun, Sumera Afzal Khan, Amjad Iqbal, Badshah Islam, Farooq Shah, Muhammad Aaqil Khan, In-Jung Lee

**Affiliations:** 1 Centre of Biotechnology and Microbiology, University of Peshawar, Peshawar, Pakistan; 2 Department of Botany, Abdul Wali Khan University Mardan, Mardan, Pakistan; 3 Department of Agriculture, Abdul Wali Khan University Mardan, Mardan, Pakistan; 4 School of Applied Biosciences, Kyungpook National University, Daegu, Republic of Korea; Universita degli Studi di Milano-Bicocca, ITALY

## Abstract

Biodegradation of hazardous pollutants is of immense importance for maintaining a clean environment. However, the concentration of such contaminants/pollutants can be minimized with the help of microorganisms that has the ability to degrade the toxic pollutants into non-toxic metabolites. In the current study, 23 bacterial isolates were purified from the rhizospheric soil of *Sysimbrium irio*, growing as a wild plant in the vicinity of gas filling stations in Peshawar city. The isolated strains were initially screened on solid nutrient agar and further purified by culturing it on anthracene amended mineral media (PNR). The bacterial growth and anthracene disappearance were observed by calculating optical density (OD). The isolates showed a concentration-dependent growth on anthracene amended PNR media at 30°C and pH7. Also, an increase in bacterial OD from 0.351 to 1.80 with increased shaking speed was noticed. On the contrary, alternate carbon sources (glucose, fructose, sucrose) or nitrogen sources (KNO_3_, NaNO_3_, NH_4_NO_3_ and CaNO_3_) posed inhibitory effect on bacterial growth during anthracene degradation. The recorded efficiency of anthracene degradation by the selected bacterial isolate (1.4×10^23^ CFUmL^-1^ and 1.80 OD) was 82.29%, after 120 h of incubation. The anthracene was degraded to 9, 10, dihydroxy-anthracene and anthraquinone, detected through GC-MS. The efficient bacterial isolate was identified as S_13_, a new strain of *Bacillus cereus*, using 16S rRNA analysis, showing 98% homology. The isolated bacterial strain S_13_ may be used as a potential tool for bioremediation of toxic hydrocarbons and to keep the environment free from PAH pollutants.

## Background

A major portion of petroleum mixture contains fuse-ringed aromatic compounds, the polyaromatic hydrocarbons (PAHs). The PAHs are the most abundant contaminants in the atmosphere and are kept on top of the pollutants list by US Environmental Protection Agency [1, 2]. These contaminants can be found abundant around the industrial sites, such as gas production sites and wood preservation industries, and release through automobile exhausts [3] that are consistent threats to human. PAHs along with their derivatives are the major factors causing anaemia, asthma, splenomegaly and various types of cancer in humans [4]. Some of the PAHs with low molecular weights have caused reproductive abnormalities and even death in aquatic animals [5, 6].

Anthracene is used as a signature compound for the detection of PAHs contamination, as it is an integral part of many carcinogenic PAHs. Due to its hydrophobicity and potential to bioaccumulate, it is used as model compound [7] to define factors affecting bioavailability and rate of degradation of PAHs in environment. In spite of structural identity between anthracene and phenanthrene regarding the number of aromatic rings, anthracene differs in degradation due to the hydrophobic nature [4, 8, 9]. The presences of these compounds in the environment have to be taken seriously, because of their broad toxic effects on living organisms [3, 10]. PAHs in contaminated soils must be treated to avoid any possible noxious effect on environment and human health. In this regard, microbes can play key role in degradation/bioremediation of the toxic PAHs into non-toxic compounds [4].

From the last few decades, bioremediation is getting importance day by day, because it is cheap, feasible and safe to clean the contaminated localities [6, 11]. The efficient microbial strains, unlike physical and chemical treatments can completely mineralize the PAHs present on the soil surface or soil sediments [11]. Previously, it has been discovered that certain starins of fungi, bacteria and algae can feed on the harmful PAHs and produce harmless compounds out of them [12]. Haleyur, Shahsavari (13) has demonstrated that some of the microorganisms (including, *Rhodococcus* sp., *Achromobacter* sp., *Oerskovia paurometabola*, *Pantoea* sp., *Sejongia* sp., *Microbacterium maritypicum* and *Arthrobacter equi*) exhibits catechol 1,2-dioxygenase activity. The presence of catechol 1,2-dioxygenase activity enables the above mentioned microorganisms to grow on PAHs and degrade catechol [13]. Alfaalfa, rape, vetch, mulberry and mustard rhizoremediation have been reported to grow and flourish in PAHs rich soil. In fact, this might be due to the presence of extensive population of microbes, mainly PAHs degrading bacteria around the roots of those plants [14–18], which enables them to grow normally. Also, pea straw has been found to be one of the most useful sources in decreasing PAHs concentration in the soil [19]. Metagenomic analysis proved that the effect of pea straw was indirect. In fact, pea straw has biostimulated the PAHs biodegrader (*Pseudoxanthomonas* spp. and *Alcanivorax* spp) in the soil that were lying latent prior to the addition of pea straw to the soil [19]. The ability of many microorganisms to degrade hydrocarbons [20–22] through metabolism or co-metabolism have been reported over the years [5]. However, there is still a space to discover microbial strains that can serve as a potential source to be used in bioremediation of PAHs. The present study was also focused on the exploration of prospective strains of beneficial microorganism that can degrade toxic chemicals and provide stress free environment. For this purpose research was conducted to (a) isolate Anthracene degrading rhizospheric bacteria from soil; (b) identification of potential PAHs degrading strain(s) in contaminated soils.

## Methods

Nutrient agar media and mineral salts media were used for initial isolation and screening of rhizospheric bacteria. The composition of PNR and PNRG (PNR+5 mM glucose) per liter of distilled water [23, 24], is PN (20x) 50 mL used as 50 mLL^-1^: KH_2_PO_4_ 13.6% (wv^-1^), (NH_4_)_2_SO_4_ 2.4% (wv^-1^), NaOH 2.5% (wv^-1^) and R salts used as 7 mLL^-1^, MgSO_4_.7H_2_O 8% (wv^-1^), FeSO_4_.7H_2_O 0.2% (wv^-1^), HCl 0.4% (wv^-1^), Agar (2%) was used as solidifying agent.

### Collection of samples

*Sysimbrium irio* was found in a wild, i.e. growing in oil contaminated soil at an operational gas filling station in Peshawar. The texture of the soil was sandy loam with pH = 7.0 ± 0.2; soil moisture = 9.6%, maximum water holding capacity = 43.9% and electrical conductivity of 3.24 ds m^-1^, respectively. The plants from the contaminated soil were dug as a whole, identified and placed in plastic zipper bag. The samples were immediately brought to the microbiology research laboratory at the University of Peshawar and stored at 4°C till further processing.

### Isolation of bacteria

Soil samples were sieved through 2 mm mesh to collect uniform sized sample. After grading, 1 g of soil sample was serially diluted in a distilled water and then followed the spread plate method as described by Alias S [25]. The plates were incubated at 28°C for 3–4 days until appearance of the colonies. The developed colonies were carefully picked and further cultured in a fresh nutrient agar plates, the inoculation step was repeated till achieving the pure culture. After obtaining the pure cultures, they were stored in anthracene slants at 4°C.

### Screening of the isolated strains on solid media

The purified bacterial isolates were screened for their capability to utilize anthracene as a sole source of carbon and energy (required for biodegradation). A confirmatory spray-plate assay was used to check the efficiency of isolated bacterial strains to grow on media supplemented with anthracene [26, 27]. Anthracene was initially dissolved in acetone and sprayed on the plates containing bacterial culture. Acetone was then allowed to evaporate leaving anthracene on the surface of the plates to be digested by the bacteria.

### Screening of the isolated strains in liquid media

Screening in liquid medium was performed using 250 ml flasks containing 100 ml PNR media, 10% of bacterial inoculum and 1000 mgL^-1^ anthracene. The media was incubated at 28°C and the bacterial growth was monitored at every 24 h interval till 120 h. Spectrophotometric analysis of bacterial growth and disappearance of anthracene was observed in PNR media at 600 nm and 540 nm, respectively.

### Optimization of growth conditions for the isolated strains

Different parameters, like concentration of anthracene, incubation temperature and pH of the growth media were optimized. Range of anthracene concentration was (100, 150, 500 and 1000 ppm), temperature (28, 30, 35, 40, 45 and 50°C) and pH used were (4, 5, 6, 7, 8 and 9). Effect of shaking speed and inoculum size was quantified using speed of (0, 120, 150, 180, 200 and 220 rpm) and (0, 8, 9, 10, 11, 12, 13%). Alternate carbon source than anthracene used were different sugars, like sucrose, glucose and fructose for their effect on the growth of bacteria at the expense of selected PAH [28, 29]. The bacterial isolate S_13_ inoculum was exposed to UV-light for 15 minutes and added to media containing different concentrations of anthracene [30]. After every 24 h, one ml of culture broth was aseptically collected to check OD, while one ml was collected and stored at 4°C to test for degradation capability. CFU was calculated at 24 h interval till 120 h in order to check the viability of the bacterial isolate. All the experiments were performed in triplicate.

### Biodegradation experiment

The biodegradation experiment was performed using 250 ml flasks containing 100 ml PNR media, 10% of bacterial inoculum and 1000 mgL^-1^ anthracene dissolved in acetone. Acetone was allowed to evaporate, 100 mL media was poured to the flask containing different concentration of anthracene and 10% bacterial inoculum was added. The flasks were incubated at 30°C and 1 mL of sample was drawn for HPLC analysis after 24 hours interval for 5 days [31].

### Extraction of anthracene for GC-MS analysis

For GC-MS analysis, Shimadzu fused silica capillary column was used. The column temperature was set to 100°C for 1 min, 15°C min^-1^ to 160°C and 5°Cmin^-1^ to 300°C for 7min. The GC injector was held isothermally at 280°C with a splitless period of 3 min. Helium was used as the carrier gas, at a flow rate of 1 mL min^-1^ by using electronic pressure control. The GC–MS interface temperature was maintained at 280°C [32].

### Plasmid curing, isolation and agarose gel electrophoresis

Plasmid DNA was isolated from 18–24 hours old culture grown in nutrient broth. For curing experiment the culture was exposed to high temperature of 45°C and DNA isolation was done according to standard protocols as described earlier [33, 34].

### DNA isolation, molecular identification and phylogenetic analysis of S_13_

Isolation of genomic DNA was carried out using standard phenol/chloroform extraction protocol [35, 36]. Isolated DNA was run on agarose gel to check its purity. It was stored at -4°C till further use [37]. Bacterial primers cloning of nearly full length 16S rDNA and sequencing were performed according to the methods described previously [37, 38]. The 16S rRNA gene sequence of the strains was analysed at NCBI (National Centre for Biotechnology Information) using BLAST tool and compared to the corresponding neighbour sequences from GenBank-NCBI database. Consensus sequence was imported into the Multalin program and multiple alignments were performed with related species (GenBank-NCBI database). Sequences were compared to those present in the data bank using blast and aligned with the ClustalW program. The results obtained were further imported into the MEGA-7 software for the construction of a phylogenetic tree using Bootstrap analysis and maximum likelihood with 500 replicates, the substitution method used was the Kimura 2-Parameter model and the statistical method used was maximum likelihood [39].

## Results

### Isolation of bacteria from collected samples

A total of 25 bacterial strains were isolated from rhizospheric soil samples collected from *S*. *irio*. Theses strains were cultured on solid and liquid media amended with anthracene.

### Screening of isolated strains on anthracene amended solid media

Out of 25 bacterial isolates grown on anthracene amended nutrient agar media, 23 strains were found to utilize anthracene as a main source for energy, when cultured on PNR media amended with anthracene (Table 1).

**Table 1:** Screening of bacterial isolates from *S*. *irio* on anthracene amended PNR media.

No	Isolate	Anthracene Concentration in ppm													
		25	50	100	200	300	400	500	600	700	800	900	1000	1100	1200
1.	S_2_	+++	+++	+++	+++	+++	+++	+++	+++	+++	+++	+++	+++	+++	+++
2.	S_5_	+++	++	+++	+++	+++	+++	+++	+++	+++	+++	+++	+++	+++	+++
3.	S_6_	+++	++	+++	+++	+++	+++	+++	+++	+++	+++	+++	+++	+++	++
4.	S_7_	+++	++	+++	+++	+++	+++	+++	+++	+++	+++	+++	+++	+++	++
4.	S_11_	+++	++	+++	+++	+++	+++	+++	++	+	+	+	-	-	-
5.	S_12_	+++	+++	+++	+++	+++	+++	+++	++	+	+	+	-	-	-
6.	S_13_	+++	++	+++	+++	+++	+++	+++	+++	+++	+++	+++	+++	+++	+++
7.	S_15_	+++	+++	+++	+++	+++	+++	+++	++	+++	++	+	+	-	-
8.	S_16_	+++	+++	+++	+++	+++	+++	+++	+++	+++	++	++	++	+	+
9.	S_18_	+++	+++	+++	+++	+++	+++	+++	++	+++	++	++	++	+	+
10.	S_19_	+++	+++	+++	+++	+++	+++	+++	+++	++	++	++	++	++	+
10.	S_20_	+++	+++	+++	+++	+++	+++	+++	+++	++	++	++	++	++	++
11.	S_21_	+++	++	+++	+++	+++	+++	+++	++	++	++	+	+	+	-
12.	S_22_	+++	+++	++	+++	+++	+++	+++	+++	++	+	+	+	-	-
13.	S_23_	+++	+++	+++	+++	+++	+++	+++	++	++	++	+	+	-	-
14.	S_24_	+++	++	+++	+++	+++	+++	+++	+++	+++	+++	+++	++	+	-
15.	S_30_	+++	++	+++	+++	+++	+++	+++	+++	+++	+++	++	++	+	-
16.	S_31_	+++	+++	+++	+++	+++	+++	+++	++	++	++	++	+	+	+
17.	S_33_	+++	++	+++	+++	+++	+++	+++	+++	+++	++	++	-	-	-
18.	S_34_	+++	+++	+++	+++	+++	+++	+++	+++	+++	+++	++	+	+	+
19.	S_36_	+++	+++	+++	+++	+++	+++	+++	+++	+++	+++	++	+	-	-
20.	S_37_	+++	+++	+++	+++	++	++	++	+	+	+	+	-	-	-
21.	S_39_	+++	+++	++	+++	+++	+++	+++	+++	+++	+++	+++	++	+	+
22.	S_40_	+++	+++	+++	+++	+++	+++	+++	+++	+++	++	++	+	+	-
23.	S_51_	+++	+++	+++	++	++	++	+	+	+	+	+	-	-	-

+++ = Rich growth

++ = Medium growth

+ = Less growth

(-) = No growth

### Screening of isolated strains in liquid media

**Fig 1:**
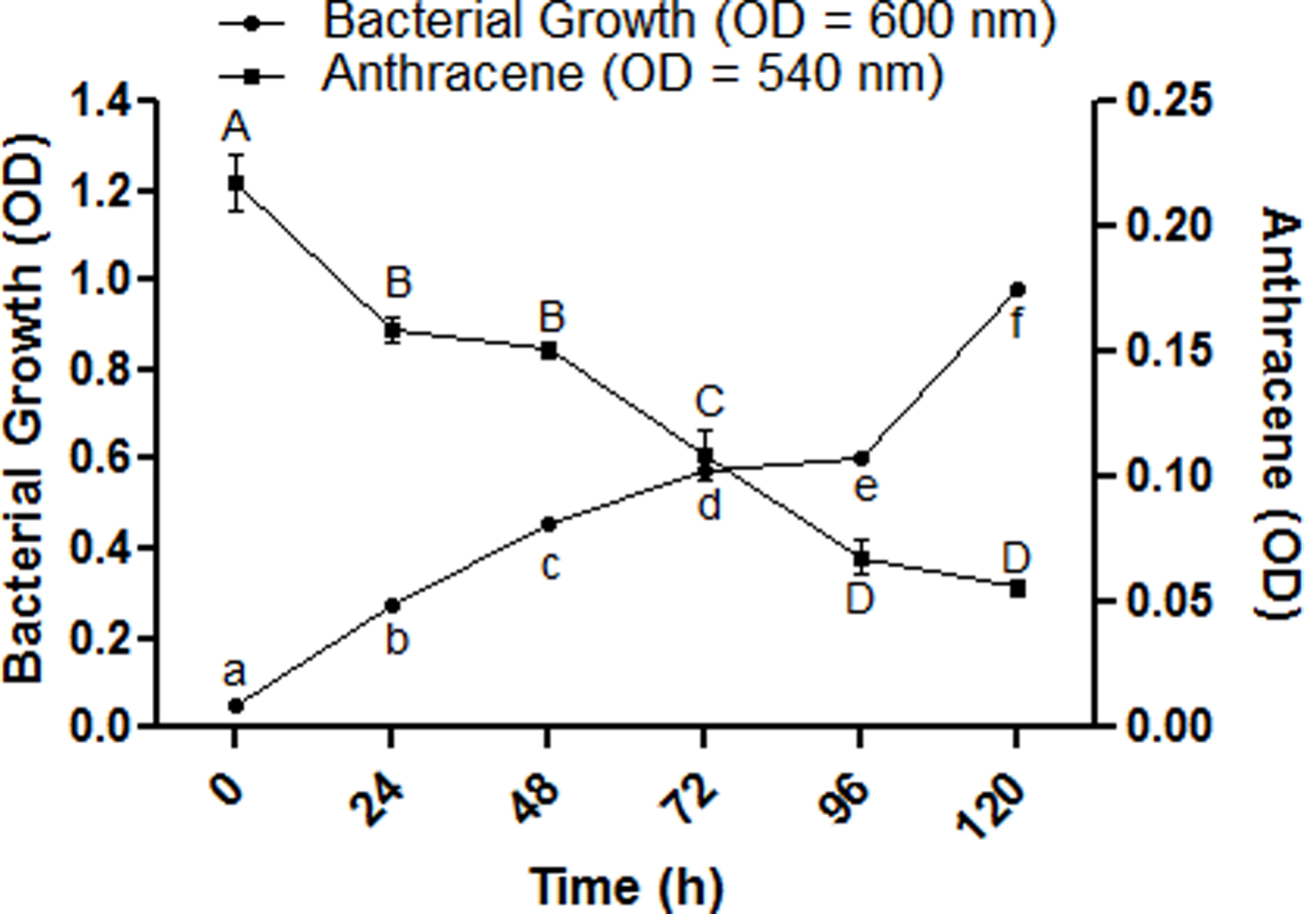
UV-spectrophotometric analysis of bacterial growth and anthracene disappearance. The OD of bacterial growth was observed at 600 nm; the OD of anthracene concentration was observed at 540 nm. Each data point represents the mean of triplicated data with ±S.E. The data points with similar letters are not significantly different at P < 0.05.

The isolates that performed best on PNR-anthracene media were further screened in liquid media. Out of 23 bacterial isolates 12 isolates having highest OD were selected for further study (Table 2). In order to confirm the bacterial growth (24 h interval till 120 h) at the expense of anthracene in liquid media, spectrophotometric analysis of bacterial growth and disappearance of anthracene was observed in PNR media at 600 nm and 540 nm, respectively (Fig 1). The results of an optimization study of parameters including anthracene concentration, temperature, pH of the media, alternate carbon and energy source, effect of nitrate salts and UV-light, shaking speed and inoculum size are given below.

**Table 2:** Bacterial growth on anthracene after 3-days incubation in large test tube (600 ppm) PNR.

S. No	Strain	OD_600nm_
1.	S_2_	0.199
2.	S_5_	0.321
3.	S_6_	0.211
4.	S_12_	0.159
5.	S_13_	0.425
6.	S_15_	0.200
7.	S_19_	0.269
8.	S_24_	0.223
9.	S_30_	0.265
10.	S_31_	0.169
11.	S_34_	0.243
12.	S_51_	0.231

### Optimization of anthracene concentration and bacterial inoculum

**Fig 2:**
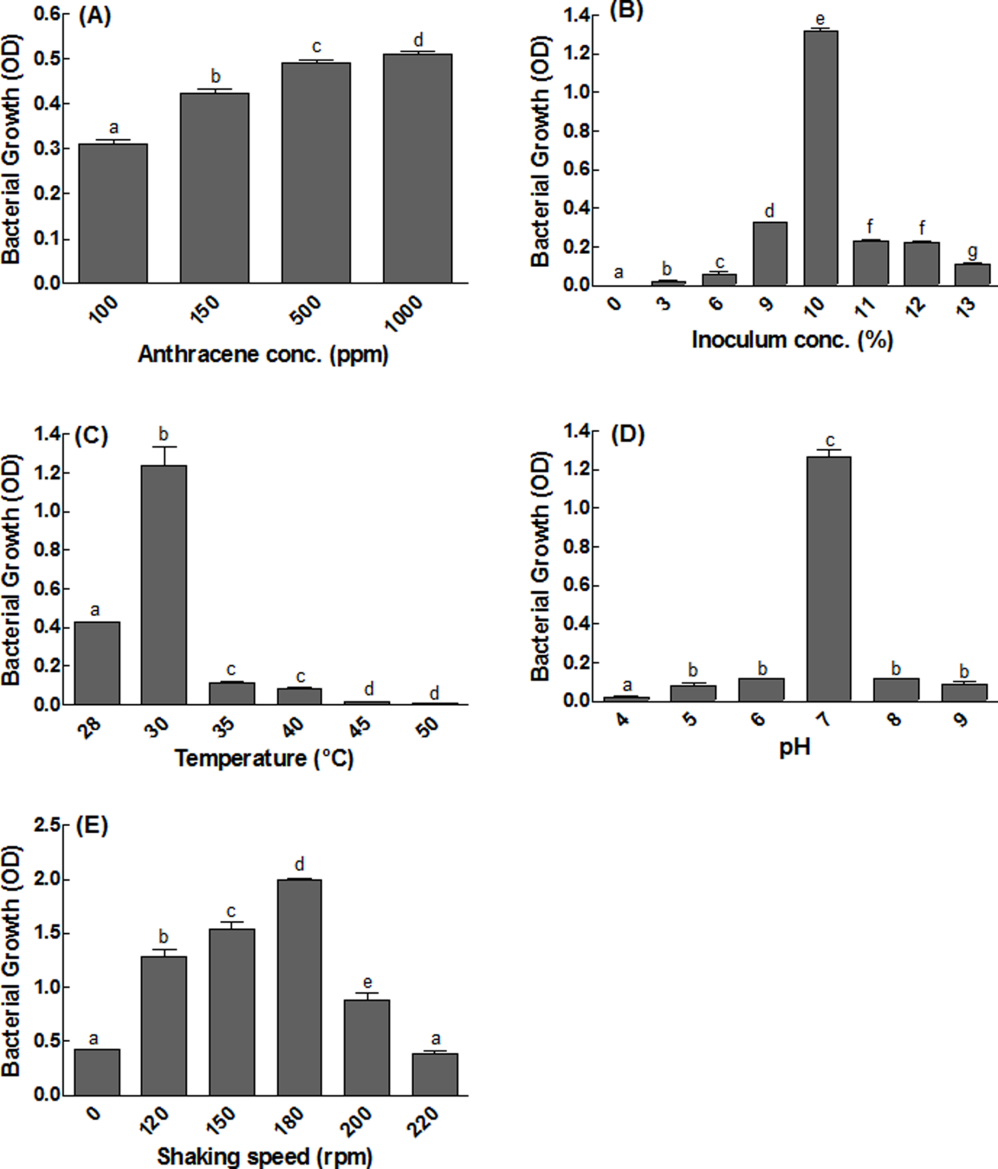
Optimization of conditions for the growth of bacterial isolates S_13_. (A) represents optimization of anthracene concentration vs growth of isolate S_13_; (B) represents optimization temperature vs growth of isolate S_13_; (C) represents optimization of media pH vs growth of isolate S_13_; (D) represents optimization of agitation speed vs growth of isolate S_13_; (E) represents optimization of inoculum concentration (%) vs growth of isolate S_13_. Each bar represents the mean of triplicated data with ±S.E. The bars with similar letters are not significantly different at P < 0.05.

The optimized concentration of anthracene was 1000 ppm for isolate S_13_ during this study (Fig 2A) and inoculum concentration was 10% vv^-1^ for maximum degradation as shown in Fig 2B.

### Optimization of temperature, pH and shaking speed for anthracene degradation

Maximum growth of bacterial isolate S_13_ was observed at 30°C (Fig 2C), at optimized pH 7 as shown in Fig 2D. Maximum growth was observed at 180 rpm as shown in Fig 2E.

### Effect of different carbon and nitrogen sources on growth of isolated S_13_

**Fig 3:**
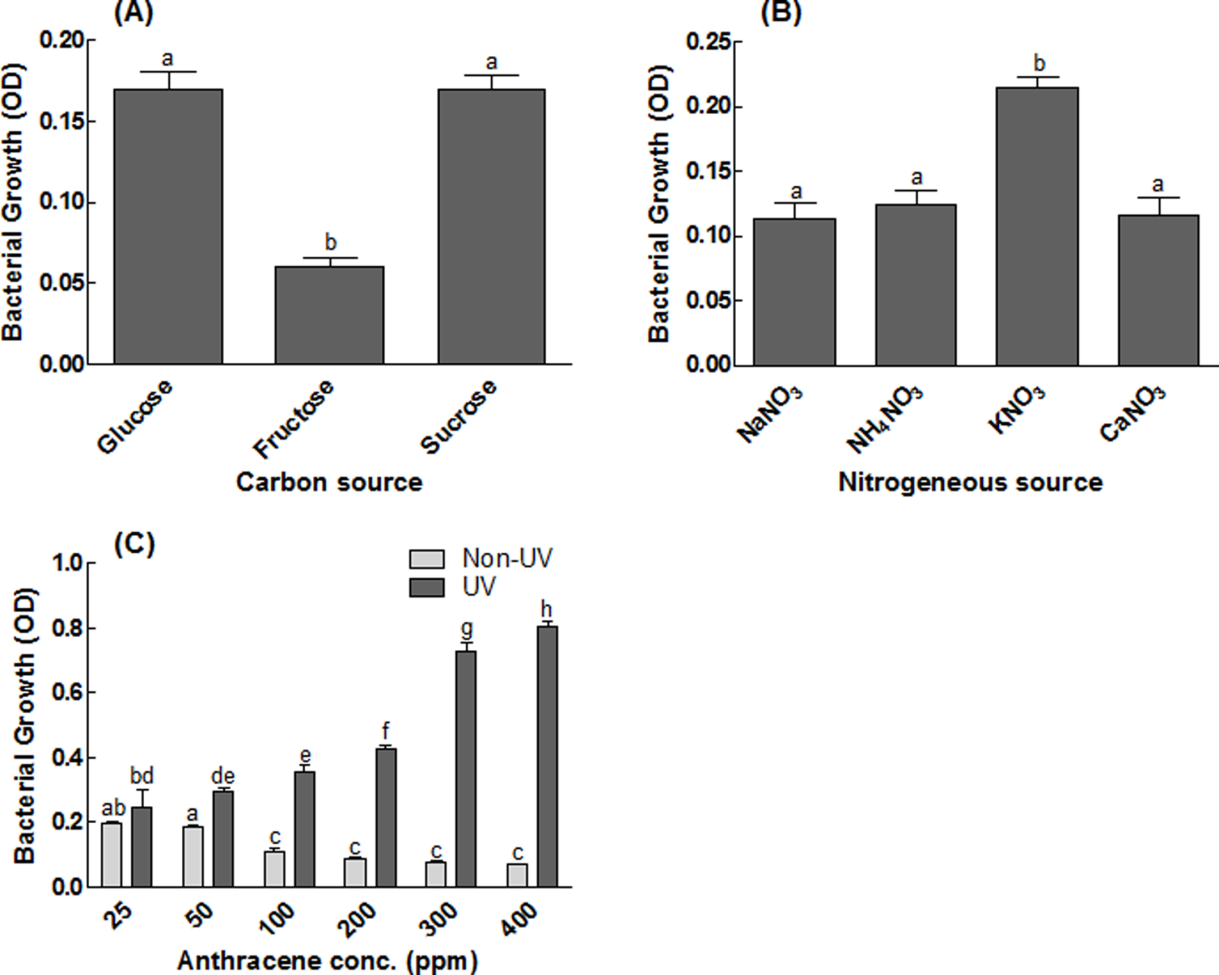
Effect of UV and different media on the growth of of bacterial isolate S_13_. (A) represents the effect of different carbon sources on the growth of isolate S_13_; (B) represents the effect of different nitrogen source on the growth of isolate S_13_; (C) represents the effect of UV light and anthracene concentration on the growth of isolate S_13_ incubated for 96 h. Each bar represents the mean of triplicated data with ±S.E. The bars with similar letters are not significantly different at P < 0.05.

Both the carbon sources and nitrate salts used were inhibitory on the isolate growth except potassium nitrate, with enhanced growth of our isolate (Fig 3A and 3B).

### Effect of UV-light induced mutation on anthracene utilization

The UV-light treated S_13_ isolate gave better growth results with increasing concentration of anthracene as compared to control experiment, not exposed to UV-light, as shown in Fig 3C.

### CFU mL^-1^ of isolated bacteria

The viability of bacteria was determined by a CFU study of samples drawn after every 24 h intervals for 120 h as shown in Table 3. The cells showed an increase in growth from initial 1.5×10^6^ per ml to 1.4×10^23^ after 120 h.

**Table 3:** CFUmL^-1^ of the bacterium isolate S_13_.

Time (h).	0	24	48	72	96	120
CFUmL^**-1**^	1.5×10^6^	2.9×10^11^	3.8×10^14^	1.8×10^17^	3.2×10^20^	1.4×10^23^

### Biodegradation of anthracene

**Fig 4:**
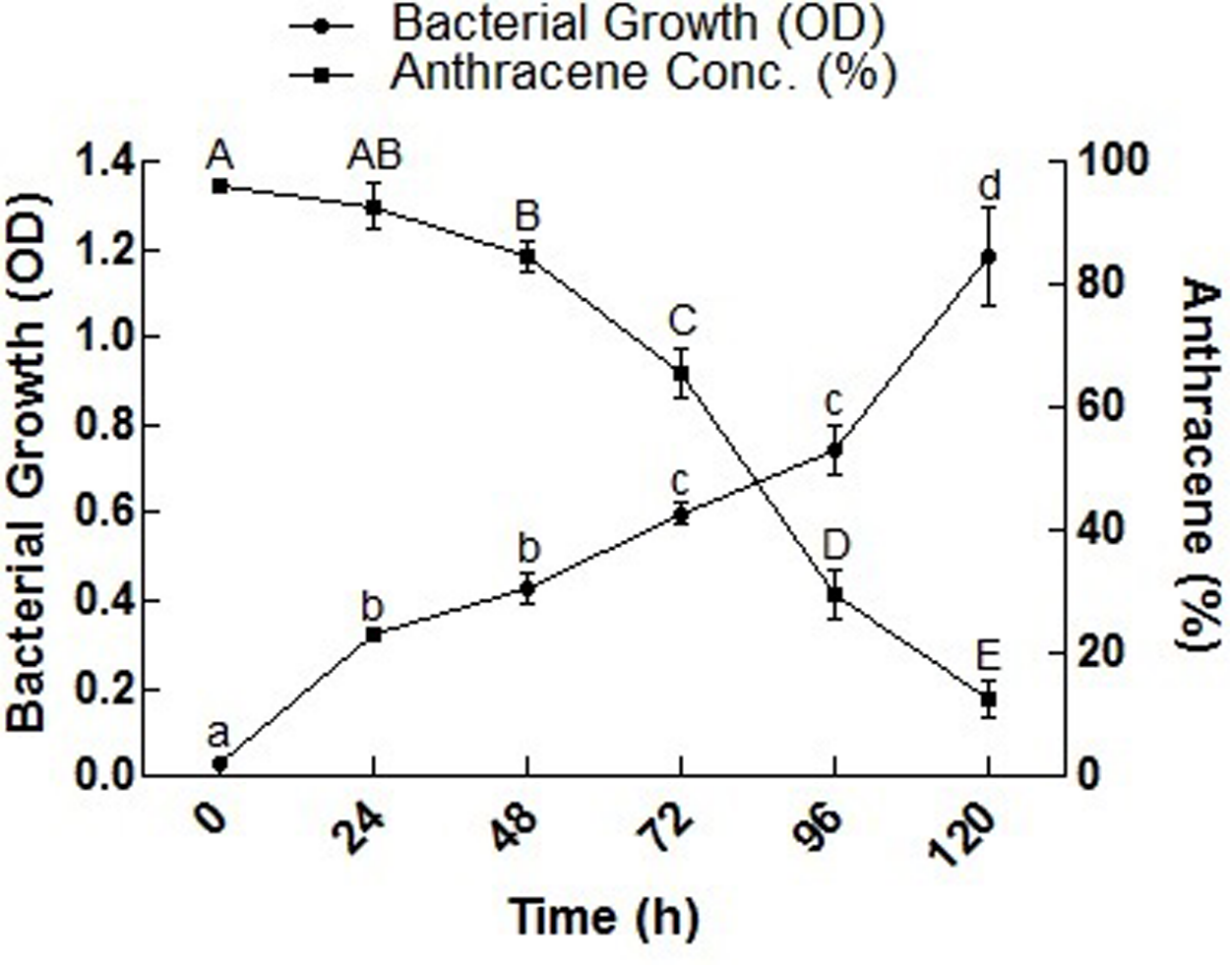
Anthracene disappearance by bacterial isolate S_13_. Each bar represents the mean of triplicated data with ±S.E. The bars with similar letters are not significantly different at P < 0.05.

**Fig 5:**
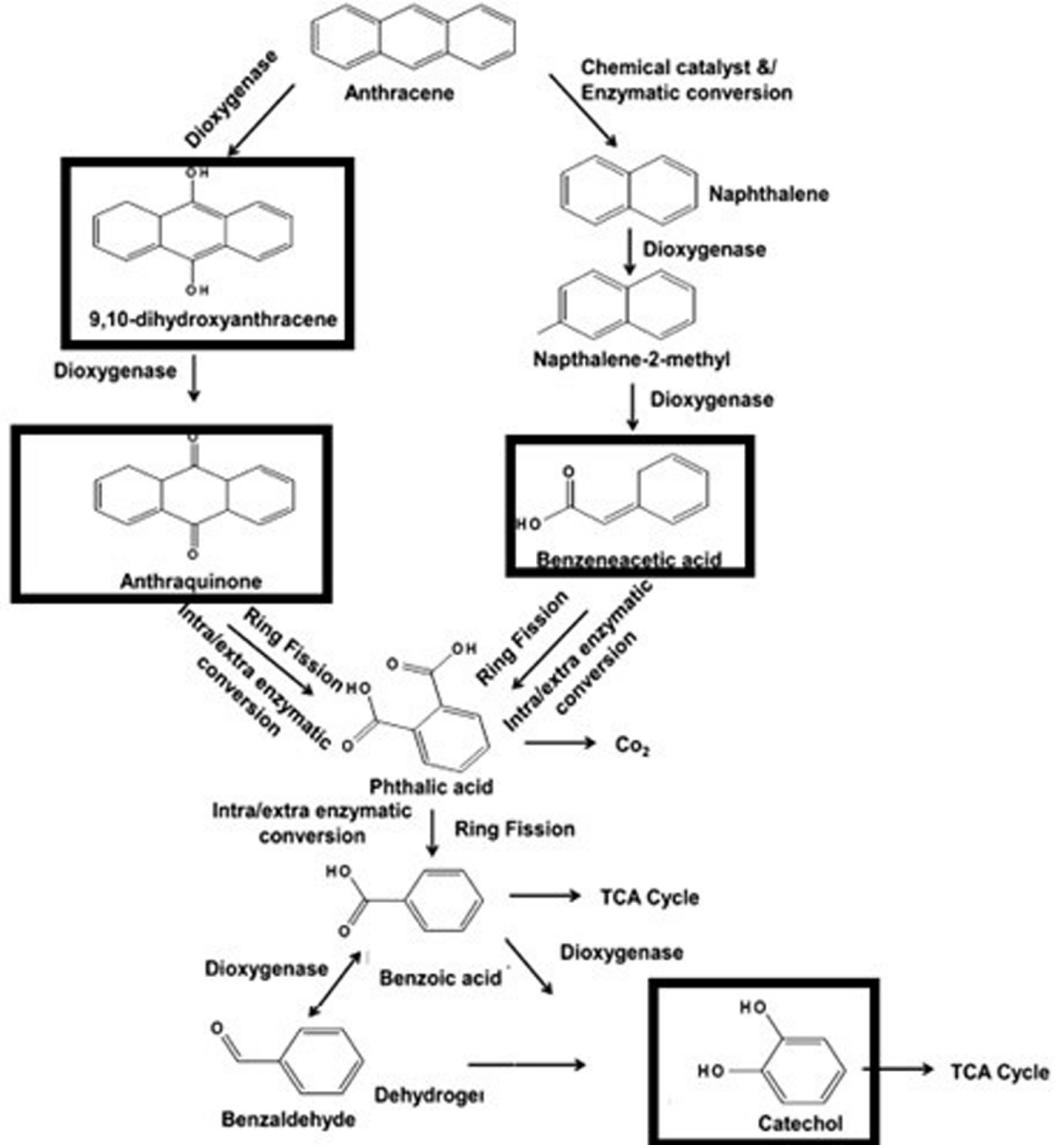
Biodegradation pathway of anthracene. Bold squares show the intermediates detected using GC-MS analysis of biodegraded samples by bacterial isolate S_13_.

Biodegradation of anthracene and bacterial growth OD are shown in Fig 4. The isolate S_13_ degraded 82.29% anthracene in 120 h. Strain S_13_ degraded the anthracene effectively during the incubation period of 24 to 96 h, whereas the growth of the bacterial isolate reached to its maximum (OD = 1.15) at 120 h. Increase in the growth of S_13_ was observed with an OD value of 0.598–1.15 between 96–120 h at the expense of only 36.19% anthracene. Confirmation of anthracene biodegradation by bacterial isolate S_13_ was sought by GC-MS analysis. The identified products included 9, 10-dihydroxyanthracene, anthraquinone, benzene acetic acid and catechol, respectively (Fig 5).

### Plasmid curing and isolation

**Fig 6:**
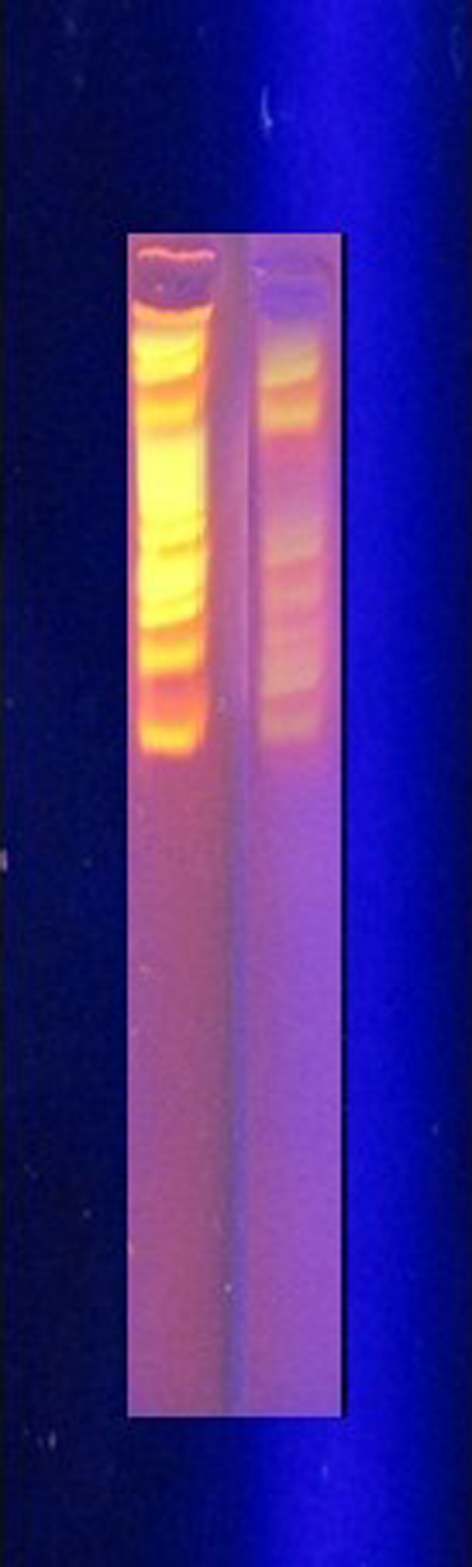
Plasmid bands from bacterial isolate S_13_. 1 Kb ladder is on the left side, whereas S_13_ plasmid is on the right side of the figure.

**Fig 7:**
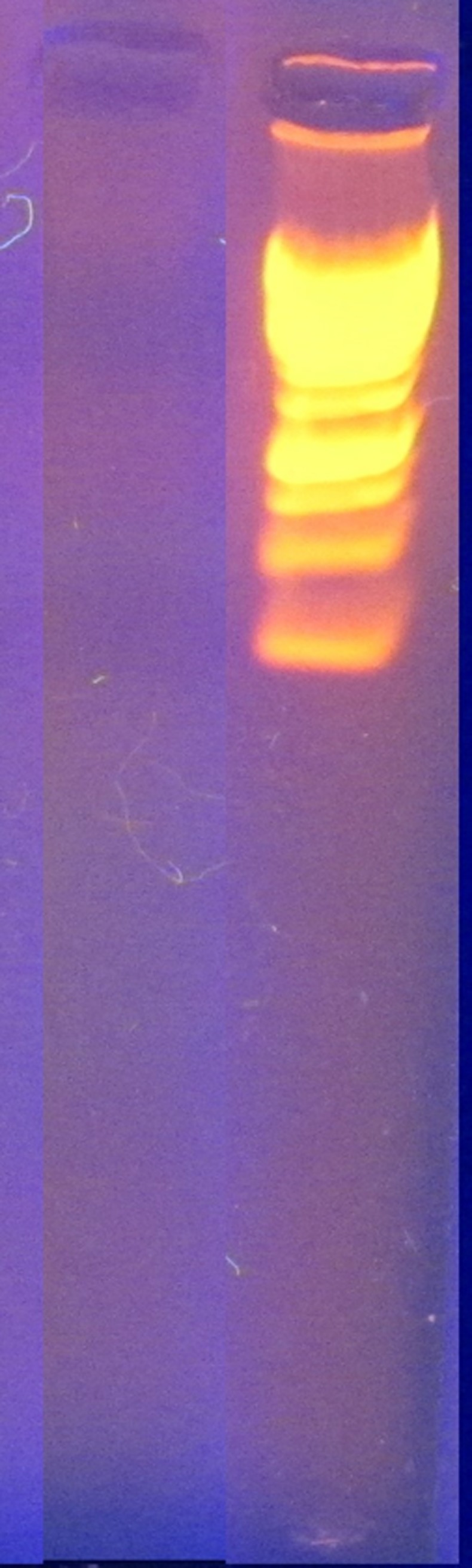
Cured plasmid sample from bacterial isolate S_13_ against 1 Kb ladder. No band can be seen (Left).

**Fig 8:**
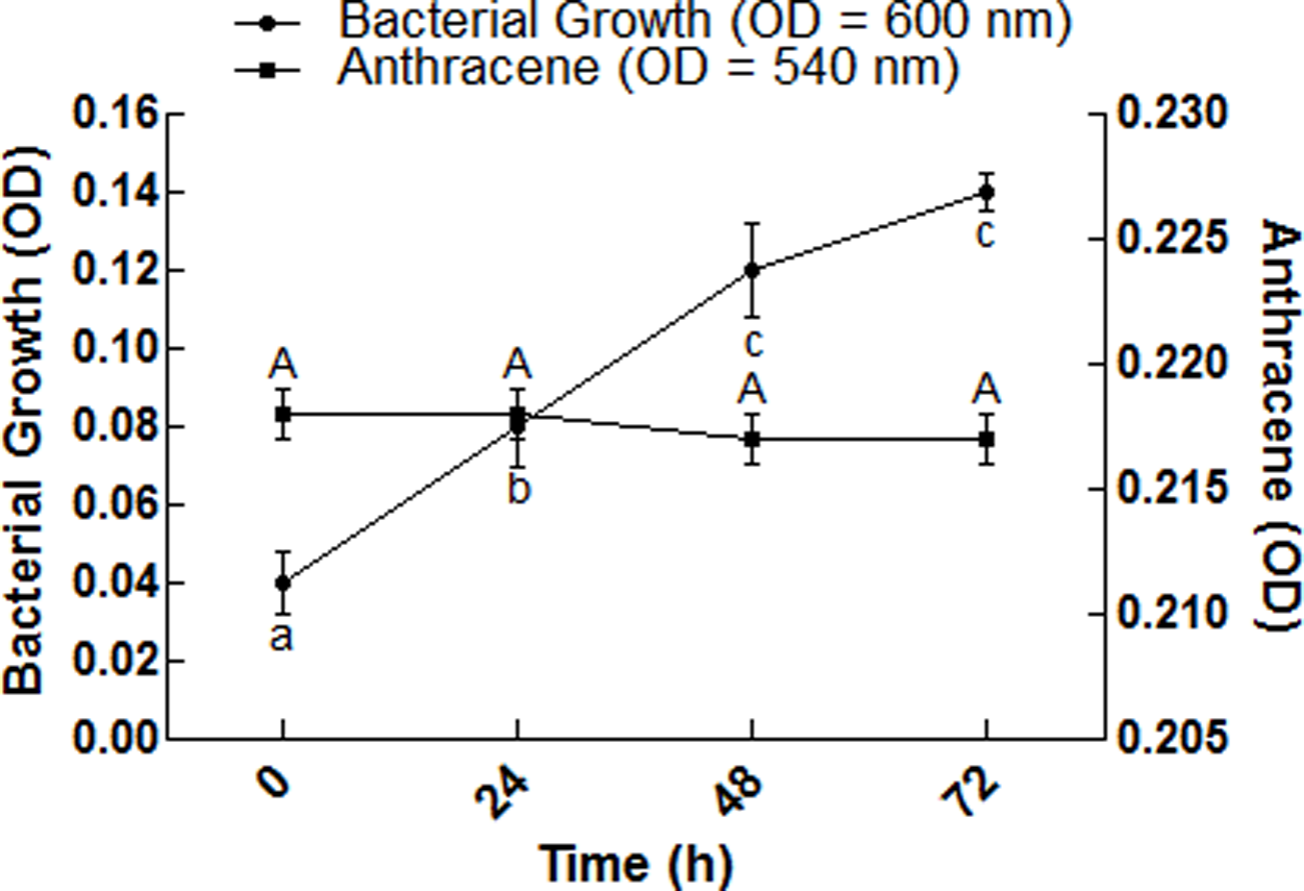
Degradation of anthracene by bacterial isolate S_13_ after plasmid curing. The OD of bacterial growth was observed at 600 nm; the OD of anthracene concentration was observed at 540 nm. Each data point represents the mean of triplicated data with ±S.E. The data points with similar letters are not significantly different at P < 0.05.

The results from agarose gel electrophoresis and plasmid curing suggested that anthracene degradation is certainly plasmid associated. The isolated plasmid from bacterial culture without curing treatment and gel electrophoresis indicated the presence of 7 plasmid bands of different sizes (Figs 6 and 7). Cured culture gave negative results for the plasmid presence and was unable to grow on anthracene amended media (Fig 8).

### Identification of bacterial isolate S_13_

Initially the bacterial isolate was identified biochemically. The result showed it was gram positive, motile, rod shaped, catalase and urease positive and was capable of starch hydrolysis, while it was negative for citrate, casein hydrolysis and indole production (Table 4). Colony morphology on agar plate showed off white/creamy color colonies with irregular margins, thus identified as *Bacillus sp*.

**Table 4:** Biochemical tests for the identification of isolate S_13_.

Microscopy	Biochemical tests				
Rods	Gram’s Test	Catalase	Starch hydrolysis	Citrate	Urease
	+ive	+ive	+ive	+ive	+ive

### Molecular identification and phylogenetic analysis of isolate S_13_

**Fig 9:**
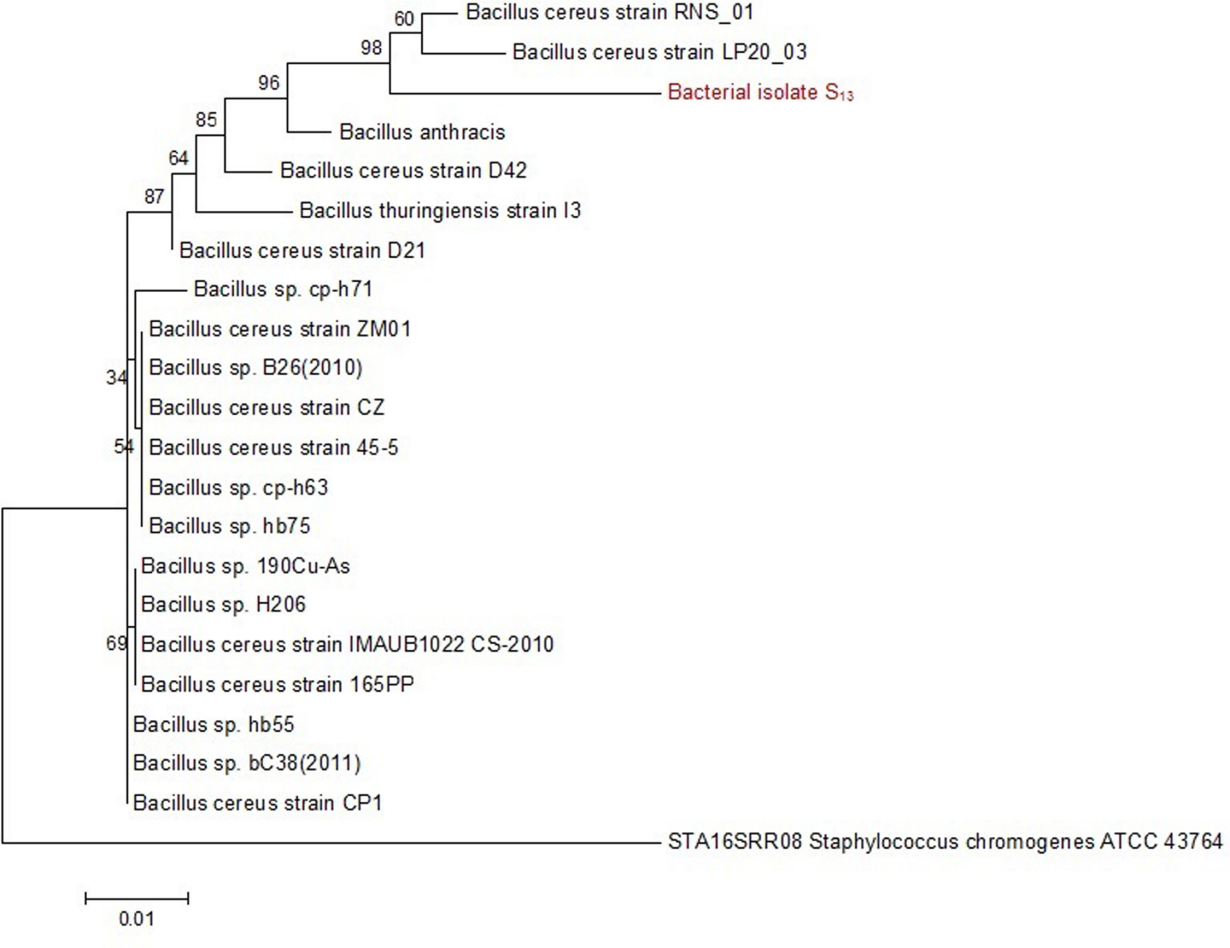
Phylogenetic analysis of strain S_13_. The evolutionary history was inferred by using the Tamura-Nei model Maximum Composite Likelihood (MCL) approach and then selecting the topology with superior log likelihood value. The tree is drawn to scale, with branch lengths measured in the number of substitutions per site. Evolutionary analyses were conducted in MEGA7.

The strain isolated from the rhizospheric soil samples collected from *S*. *irio* was identified by ITS rDNA region sequencing analysis. Phylogenetic analysis was carried out through MEGA 7.0 software for the construction of a phylogenetic tree using Bootstrap analysis and maximum likelihood with 500 replicates. A total of 20 sequences were downloaded from BLAST data that were showing the maximum relatedness with our isolate. Results of BLAST search showed highest sequence similarity (98%) between the bacterial isolate S_13_, capable of anthracene utilization as carbon and energy and *Bacillus cereus* RNS-1, *Bacillus cereus* strain LP20-03. The strain also showed 87% similarity with *Bacillus thurengensis* strain 13. On the basis of sequence homology and phylogenetic analysis, the isolated bacterial strain was identified as S_13_ strain of *B*. *cereus* (Fig 9).

## Discussion

Polycyclic aromatic hydrocarbons are the main concern for the world environment that causes great damage to humans, plants and animals wellbeing. In the current study, we have identified an isolate S_13_ from rhizospheric soil samples collected from *S*. *irio* with high activity against anthracene. Furthermore, the observed bacterial isolate had achieved higher growth at an increased level of anthracene from 100–1000 ppm. Similar results have been reported in the past where higher growth was attained by the bacterial strain in a medium enriched with anthracene [31]. Other factors that can be detrimental to bacterial growth and activity to digest PAHs include temperature, pH, aeration and the presence of nutrients in the medium.

Rise in temperature can affect both solubility [40] and degradation of PAHs by bacteria. For instance, high temperature can make the PAHs more soluble and bioavailable, whereas it also decreases the solubility of oxygen that can mainly affect the activity of aerobic bacteria. Therefore, most of the previous researches tend to focus on moderate temperatures rather high or low temperatures. Likewise, in the present study, high anthracene degradation has been noticed at 30°C, which can be attributed to the optimal growth conditions of the selected strain. The optimal growth conditions can allow the MO to secrete a vast array of enzymes in the surroundings that can degrade the toxic compounds in question. Unsuitable temperatures can deter the enzyme action by blocking its access to substrate due to insolubility (low temperature) or effecting the confirmation of the enzymes (high temperature).

Similarly, all MO can perform its activity at certain pH range, i.e. minimal, maximal and an optimal pH, where at optimal pH the activity of the MO is significantly high. Any drastic changes in pH can interfere with cell wall and cell contents of the MO, thus affecting its growth and metabolism [3]. The result of this study also revealed that at pH7 the growth and activity of the bacterial isolate S_13_ was high due to balanced ionic distribution inside and outside of the cells. However, changes in pH can disturb the ionic balance and disrupt the growth and metabolism of the bacterial isolate S_13_, resulting in low degradation of PAHs.

Shaking speed also proved to be an important factor in the aerobic degradation of PAHs that needed to be optimized in order to achieve optimum bacterial growth and degradation of the pollutants. Proper aeration has considerably improved the growth of S_13_ in the anthracene supplemented medium. Faster agitation could result in a higher degradation rate, which can be credited to sufficient supply of oxygen and dissolution of solute in the solvent to be taken up by microorganism [41]. Also, the degradation of anthracene by S_13_ might be facilitated by oxygenase enzyme. The level of enzyme production and activity might enhance in the presence of molecular oxygen that can lead to a complete degradation of anthracene. Certainly, ample supply of oxygen could efficiently incorporate it enzymatically in the aromatic ring of the anthracene, a rate regulating step in the biodegradation of PAHs [26, 42, 43].

Some microorganisms can consume PAHs as a source of carbon and energy [44], but the presence of glucose in the form of additional carbon source might effect it [45]. This has been confirmed by the present study, where S_13_ failed to digest the anthracene completely as a sole carbon source in the presence of glucose, fructose and sucrose. This means that supplementation of media with any alternate carbon sources can influence the degradation of anthracene. The readily available carbon sources may negate the degradation of anthracene due to prior assimilation, thus causing inhibition of enzymes responsible for anthracene degradation [46]. Correspondingly, supplementation of the growth media with different nitrogen sources (KNO_3_, NaNO_3_, CaNO_3_ and NH_4_NO_3_) has no significant effect on growth and degradation activity of anthracene. The negative effect of different energy and nitrogen sources in this study might be linked to its role as a competitor to anthracene, making anthracene less available to be degraded.

Anthracene is hydrophobic due to its cyclic structure and lack of highly hydrophilic hydroxyl groups (-OH) that might be the reason for the slow rate of biodegradation by the MO. The rate of bioremediation of a pollutant mainly depends on the number and nature of degrading organism, intrinsic and extrinsic factors, solvent and chemical structure of the compound to be degraded. Algae, fungi and bacteria have the capabilities to degrade PAHs into less complex substances through biotransformation mechanisms [16]. Though, MO needs to adapt the conditions first that allow the fast growth of microbial populations with the ability to degrade PAHs [47]. Additionally, bioavailability-induced adaptations are equally important for microbial populations to build an interaction with contaminants and make it more bioaccessible [48]. Similarly, the presence of other contaminants can affect the efficiency of microbial degradation, which is critical in terms of biodegradation and bioremediation. Despite well-studied phenomenon, there remains limited understanding of many fundamental aspects of plant-microbe interactions during PAHs phytoremediation [49, 50]. In the present study, it was noticed that 82.92% anthracene was degraded in 120 h in PNR media contrary to the past reports. Previously, 74.8% anthracene degradation was recorded in BSM media on the 10^th^ day of incubation, whereas the complete degradation of added anthracene to autoclaved soil by *Burkholderia* sp. has taken 20 days [10, 51]. In fact, the chemical structure and nature of a compound (including, molecular weight, water solubility and lipophobicity) and nature of MO would affect the bioaccumulation and the rate of degradation of the compound by MO. The results of this study have confirmed this argument, where bacterial isolate S_13_ has actively degraded anthracene (degradation rate = 82.29%) within six days, contrary to previous reports (89% degradation of three ring PAHs within seven weeks) [52, 53].

## Conclusion

*Bacillus cereus* S_13_ can be used for biodegradation of anthracene, which is the main pollutants of incomplete organic combustion produced by petroleum and coal industry. We isolated novel anthracene biodegrading bacterium. Our isolate used anthracene as a sole source of carbon and it can be utilized for bioremediation of other PAHs. *Bacillus cereus* S_13_ can be a potential tool for bioremediation of toxic hydrocarbons and to keep the environment free from PAH pollutants. Though, the development of precise and effective technology for the treatment of complex PAHs mixtures is still needed.

## Abbreviations

PAHs: Polyaromatic hydrocarbons
LMW: Low-molecular weight
OD: Optical density
HPLC: High performance liquid chromatography
GC-MS: Gas chromatography–mass spectrometry
DNA: Deoxyribonucleic acid
rRNA: Ribosomal ribonucleic acid
NCBI: National Centre for Biotechnology Information
UV: Ultraviolet light
BLAST: Basic Local Alignment Search Tool
CFU: Colony forming units
KH_2_PO_4_: Potassium phosphate monobasic
(NH_4_)_2_SO_4_: Ammonium sulfate
NaOH: Sodium Hydroxide
MgSO_4_.7H_2_O: Magnesium Sulfate Heptahydrate
FeSO_4_.7H_2_O: Iron(II) Sulfate Heptahydrate
HCl: Hydrogen chloride
KNO_3_: Potassium nitrate
NaNO_3_: Sodium nitrate
CaNO_3_: Calcium nitrate
NH_4_NO_3_: Ammonium nitrate
